# Application of spectral CT combined with perfusion scan in diagnosis of pancreatic neuroendocrine tumors

**DOI:** 10.1186/s13244-022-01282-9

**Published:** 2022-09-04

**Authors:** Yamin Wan, Hui Hao, Yunjin Chen, Yifan Zhang, Qingmei Yue, Zhizhen Li

**Affiliations:** 1 Department of Radiology, The First Affiliated Hospital of Zhengzhou University, No.1 Jianshe Road, Zhengzhou, 450052 China; 2 Department of Radiology, Zhengzhou Central Hospital, Zhengzhou, 450000 China; 3 Department of Endocrinology, The First Affiliated Hospital of Zhengzhou University, No.1 Jianshe Road, Zhengzhou, 450052 China

**Keywords:** Pancreatic neuroendocrine tumors (pNETs), Spectral CT combined with perfusion scan, Imaging diagnosis, Radiation dose

## Abstract

**Background:**

Pancreatic neuroendocrine tumors (pNETs) are heterogeneous tumors from the pancreatic neuroendocrine system, and early diagnosis is important for tumor prognosis and treatment. In this study, we aimed to explore the diagnostic value of spectral CT combined with perfusion scanning in improving the detection rate of pNETs.

**Methods:**

From December 2018 to December 2020, 58 patients with clinically suspected pNETs were prospectively enrolled in the study for one-stop spectral CT combined with perfusion scanning, 36 patients were confirmed with pNETs by histopathology. An independent cohort of 30 patients with pNETs who underwent routine pancreatic perfusion scanning in our hospital during the same period were retrospectively collected. The image characters of pNETs versus tumor-free pancreatic parenchymal were examined.

**Results:**

The detection rate of spectral CT combined with perfusion was 83.1–96.2%. CT values of the pNETs lesions under each single energy in the arterial phase were statistically higher than those of the adjacent normal pancreatic parenchyma. IC, WC and NIC, in the arterial phase of pNETs lesion were all statistically higher than those of the adjacent normal pancreatic parenchyma. The perfusion parameters of pNETs including BF, BV and MSI were significantly higher than those in normal parenchyma. The average effective radiation dose during the perfusion combined energy spectrum enhanced scanning process was 17.51 ± 2.18 mSv.

**Conclusion:**

The one-stop spectral CT combined with perfusion scan improves the detection of pNETs according to morphological features, perfusion parameters and energy spectrum characters with a relatively small radiation dose.

## Key points

- Spectral CT combined with perfusion scan improves pNETs detection based on morphological features, perfusion parameters and energy spectrum characters.
- Combined perfusion and spectral scan at the optimal keV in the arterial phase of the energy spectrum has high sensitivity and specificity in detecting pNETs.
- The radiation dose of one-stop perfusion combined energy spectrum enhanced scanning process is relatively low.

## Background

Pancreatic neuroendocrine tumors (pNETs) are heterogeneous tumors originating from pluripotent stem cells of the pancreatic neuroendocrine system. Incidence of pNETs has increased to10% of all pancreatic tumors in recent years which are no longer considered rare [1–3], partly due to the wider application of computerized tomography (CT), endoscopic ultrasonography and other imaging techniques [4].

For patients with clinically suspected pNETs, imaging examinations are of great significance for diagnosis in determining the location of lesion, presence of metastasis, tumor grade, and guiding surgery or treatment plans. Traditional enhanced multidetector CT (MDCT) imaging has been the main force to diagnose and grade pNETs [5, 6], and for most pNETs, the lesions are characterized by rich blood supply with intense and homogeneous "fast-in and fast-out" enhancement on arterial and portal phases. However, some pNETs are characterized by lack of blood supply, especially for large tumors with cystic degeneration, making them difficult to differentiate from pancreatic cancer and pancreatic cystic tumors [7]. Furthermore, a small number of pNETs have atypical manifestations such as transient or delayed enhancement; traditional enhanced MDCT showed limited sensitivity and specificity in detecting these tumors [8]. Pancreatic CT perfusion imaging can distinguish lesions from surrounding tissue structures, and combine functional information with good spatial resolution [9], which has important application value in diagnosing pNETs accurately, but its clinical application is controversial due to high radiation dose and high noise ratio [7].

As a new technology, CT energy spectrum imaging has been developed in the field of CT imaging. Through CT energy spectrum imaging, superior material differentiation is achieved using the energy dependence of X-ray attenuation, which improves the image quality, and at the same time suppresses the beam hardening artifact and reduces the radiation dose. Particularly, soft tissue contrast can be significantly improved using multi-energy spectrum characteristics; thus, it is helpful for qualitative and quantitative diagnosis of small lesions in soft tissues that are difficult to characterize by conventional CT. Studies have reported that when iodine contrast agent is selected as the base material for CT spectroscopic imaging, it can display small lesions with rich blood supply, which has a magnifying effect on the detection of small islet cell tumors and small liver cancer [10]. General Electric (GE) Revolution CT machine from GE Company (USA) has a 160 mm z-axis detector with comprehensive coverage, which can effectively suppress beam hardening artifacts and reduce radiation dose; it uses K-edge imaging to reduce radiation dose; it adopts adaptive statistical iterative reconstruction (ASiR-V) to reduce radiation dose and improve image quality. To this end, we performed a clinical study in assessing the characteristics of perfusion and energy spectral parameters of pNETs to improve tumor detection by applying the spectral CT combined with perfusion scan with GE Revolution CT.

## Materials and methods

### Patients

This study was approved by the e Ethics Committee of The First Affiliated Hospital of Zhengzhou University. All patients in this research were informed and provided written consent. 58 patients with clinically suspected pNETs were prospectively enrolled in the study for one-stop spectral CT combined with perfusion scanning from December 2018 to December 2020. Suspected pNETs were referred as patients with symptoms such as epigastric pain, pancreatitis, obstructive jaundice, intractable hypoglycemia or splenomegaly and wasting and was initially diagnosed as pNETs by experienced clinicians. The inclusion criteria were as follows: (a) the CT images of the patients did not have severe motion artifacts or any metal artifacts that affect the assessment of the lesion; (b) patients did not undergo relevant treatment affecting perfusion and energy spectrum parameters before surgery; and (c) Patients with pathologically confirmed pNETs with complete clinical information. Exclusion criteria were: (a) the patient was allergic to iodine contrast agents during pregnancy; (b) presence of severe hepatic and renal insufficiency; (c) poor vascular status of the patient and inability to tolerate high speed intravenous injection. With these criteria 36 pNETs patients were recruited. An independent cohort of 30 patients with pathologically confirmed pNETs who underwent routine pancreatic perfusion scanning in our hospital during the same period were retrospectively collected, the same inclusion and exclusion criteria applied to this cohort.

### CT imaging technique

Scans were performed on a GE Revolution CT machine. Patients received iv injection of non-ionic iodine contrast agent (350 mgI/ml, Iopamidol injection, HengRui Healthcare, Jiangsu, China). Energy spectrum combined with perfusion scan method: upper abdominal plain scan plus conventional pancreas perfusion and dual-phase enhanced energy spectrum scan (arterial phase and venous phase). Scanning range: (1) Upper abdominal CT plain scan: from the top of the diaphragm to the level of the umbilicus; (2) Perfusion scan range: the upper and lower boundaries of the pancreas (determined according to the plain scan of the upper abdomen), that is, from the level of the first hepatic hilum to the level of the duodenum; (3) Two-phase energy spectrum scanning range: from the top of the diaphragm to the level of the lower border of the liver. Scanning parameters: (1) Conventional upper abdomen plain scan: helical scan, tube voltage 120 kVp, tube current 100–450 mAs, pitch 0.992:1, scanning slice thickness 5 mm, and slice spacing 5 mm. (2) Perfusion parameters: axial scan, automatic tube voltage 100 kVp, automatic tube current 100 mAs, 50ASIR-V, and the other settings were same as the plain scan. 3) Energy spectrum enhancement parameters: helical scan, tube voltage 80 kVp and 140 kVp, high and low tube voltage was switched instantaneously (25 ms), tube current automatic adjustment, and the other settings were same as the plain scan (Table 1). The scanning phase was divided into 4 parts, the 1st and 3rd parts were perfusion inflow phase and outflow phase, of which 8 images were collected in the inflow phase and 14 images were scanned in the outflow phase; in the spectral enhancement phase, the 2nd phase was the arterial phase and the 4th phase was the venous phase, and each image was collected once; a total of 24 images were collected. After the localization image was obtained, a routine upper abdominal scan was performed first, and the parameters of the perfusion combined with energy spectrum were set. The non-ionic iodine contrast medium was injected through the anterior cubital vein indwelling needle (20G Y-II type) using a double-barrel high-pressure syringe, and then 20 ml of saline was added with bolus tracking to ensure that all of the contrast medium reached the central veins, serving as a bolus chaser to increase peak arterial enhancement. Contrast agent injection: with the use of a power injector the flow rate was 5 mL/s (depending on the patient's blood vessel tolerable flow rate) with bolus tracking, and the dose was 1 ml/Kg. The perfusion scan started 6 s after contrast injection, with 8 consecutive perfusion scans of 3 s each, followed by an energy spectrum enhancement CT scan of 3 s, then 14 consecutive perfusion scans of 1.5 s each, and when 54 s after contrast injection the last energy spectrum enhancement CT scan of 3 s. After scanning, images were reconstructed: the energy spectrum scanning range was adjusted to the perfusion range, and the original 22 perfusion images and 2 energy spectrum enhanced images were fused to reconstruct the perfusion image.

**Table 1:** Comparison of different CT technical parameters

Scan sequence	Technology	Tube voltage	Tube current	Pitch	Scanning slice thickness	Slice spacing
Plain scan	Helical scan	120 kVp	100⁓450 mAs	0.992:1	5 mm	5 mm
Perfusion scan	Axial scan	100 kVp	100 mAs, 50ASIR-V	0.992:1	5 mm	5 mm
Energy spectrum enhancement	Helical scan	80 and 140 kVp	Auto-adjustment	0.992:1	5 mm	5 mm

Conventional pancreas perfusion scan method: continuous pancreas perfusion scan after upper abdominal plain scan. The perfusion scanning adopted axial scanning, tube voltage 120 kV, current 150 mA, and pitch 1.375:1. Contrast agent was injected intravenously at 3–4.5 ml/s at a dose of 1 ml/kg, and 20 ml of saline was added at the same time; perfusion images were continuously collected for 25 times.

### Imaging analysis and measurement

Analysis and measurement of the images were performed by two independent radiologists on professional workstation GE ADW4.7, both with intermediate title or above; final decision was made by consultation with a third senior radiologist in case of disagreement; the histopathological nature of each tumor was unknown to both radiologists. Perfusion images were fused and reconstructed (thickness 1.25 mm), and then sent to the post-processing workstation for analysis and measurement. The reconstructed perfusion image was subjected to automatic motion artifact correction (CT Dynamic Registration), and then loaded into CT perfusion 4D software for pancreatic perfusion analysis. The abdominal aorta was used as the inflow artery to calculate and generate a pseudo-color map of pancreatic perfusion. A region of interest (ROI) was delineated at the normal pancreatic parenchyma to obtain the perfusion parameters of the corresponding tissue, including blood flow (BF), blood volume (BV), capillary surface permeability (capillary surface permeability), permeability of surface (PS), mean transit time (MTT), time to peak (TTP), transit time to pulse peak (transit max, Tmax), mean slope of increase (MSI), contrast agent arrival delay (idoine remaining function time 0, IRF T0). Perfusion parameters between tumor lesions and normal parenchyma as well as between different grades of pNETs were compared.

Energy spectrum images in the arterial and venous phases were loaded into the GSI viewer General software, images at the axial Mono interface 70 keV were used to create ROIs in the abdominal aorta, pancreatic lesions, and adjacent normal parenchyma, respectively. Iodine-based map, water-based map and energy spectrum curve were automatically generated, iodine concentration (IC) and water concentration (WC) of the corresponding parts, normalized iodine concentration (NIC, ratio of the IC of the lesion to that of the abdominal aorta at the same layer), and the CT value of a single energy level at 60 keV to 140 keV in the lesion and adjacent pancreatic parenchyma (with 10 keV intervals) were recorded. The longest diameter of the lesions was measured and recorded on the spectrally enhanced images. ROIs were placed at the solid part of the lesions, avoiding necrotic area, calcification, and blood vessels; the scope of ROIs were kept consistently as possible; all data were measured 3 times and averaged to reduce errors. pNETs detection rate is defined as: total number of lesions detected in imaging/total number of lesions detected in histopathology.

### Scanning radiation dose

After each scan, the system automatically generated the radiation metering parameters, recorded dose-length product (DLP, mGy × cm), and calculated effective dose (ED, mSv), ED = *k* × DLP, where *k* = 0.015 (mSv × mGy^−1^ × cm^−1^) (k is the conversion factor for adult abdominal CT examination).

### Statistical analysis

Statistical analysis of the recorded data was performed using SPSS 21.0 package (SPSS Inc, Chicago, IL). Data that conformed to the normal distribution were expressed as mean ± standard deviation, those that did not conform to the normal distribution were expressed as median (95% confidence interval). Paired sample *t* test was used to compare the difference between tumor and normal tissues. Independent sample *t* test or nonparametric test was used to compare the differences of perfusion parameters at different grade. *p* < 0.05 was considered statistically significant.

## Results

### Clinical characteristics

Thirty-six patients who underwent spectral CT combined with perfusion scanning and were histopathologically confirmed as pNETs were included, including 15 males and 21 females, with an age range of 20 to 71 years, and a median age of 52 years. 22 patients were excluded, including 16 patients were diagnosed with pancreatic cancer, 3 patients were cystadenoma, 1 patient was solid pseudopapillary and 2 patients were pancreatitis. There were 52 pathologically confirmed lesions, including 16 in the head of the pancreas, 4 in the neck, 17 in the body and 15 in the tail. Among the 30 retrospective patients, 14 were males and 16 were females, with an age range of 20 to 81 years, and a median age of 53.5 years. The number of tumors that were pathologically confirmed lesions was 37, 12 lesions were in the head of the pancreas, 4 in the neck, 8 in the body and 13 in the tail. The average longest diameter of the tumors was 15.54 ± 1.36 mm, and the longest diameter of one lesion was 47 mm.

### Detection rate of spectral CT combined with perfusion scanning

**Fig. 1:**
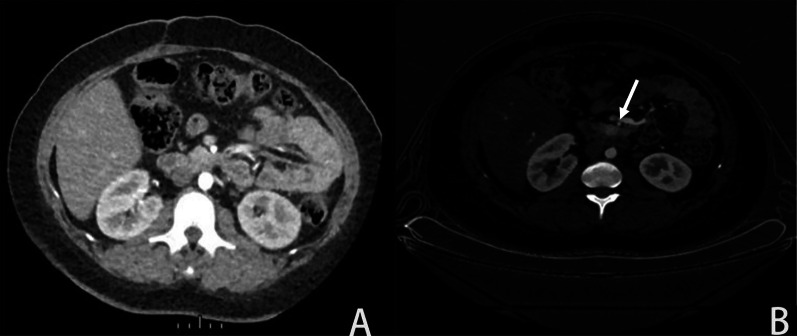
Representative pNETs image under conventional pancreas perfusion scan (**a**), in which the lesions in the uncinate process are poorly developed; the lesions are clearly displayed on the energy spectrum CT image of the arterial phase (**b**)

Most of pNETs lesions were characterized by rich blood supply, but some were not obviously enhanced and lack specificity. Some lesions were poorly visualized on conventional perfusion scans but were clearly visualized on energy spectrum (Fig. 1). The detection rate of combined perfusion and spectral scan at the optimal keV in the arterial phase of the energy spectrum was 96.6%, and that in the venous phase was 84.6%, the detection rate of spectral CT combined with perfusion was 83.1%–96.2%, and the detection rate of conventional pancreatic perfusion was 82.8%, the former was higher than the latter (*p* < 0.05). The arterial phase energy CT combined with perfusion scanning is more conducive to the detection of lesions (Table 2).

**Table 2:** Comparison of detection rate between spectral CT combined with perfusion scanning and conventional perfusion scanning

Scanning method	Conventional perfusion scanning	Spectral CT combined with perfusion scanning	
		Perfusion scanning and 70 keV single energy image	Perfusion scanning and best single energy image
Detection rate	30(37) 82.8%	47(52) 90.3% (Arterial phase)	50(52) 96.2% (Arterial phase)
		43(52) 83.1% (venous phase)	44(52) 84.6% (venous phase)

### Comparison of perfusion parameters between pNETs and adjacent pancreatic parenchyma

**Fig. 2:**
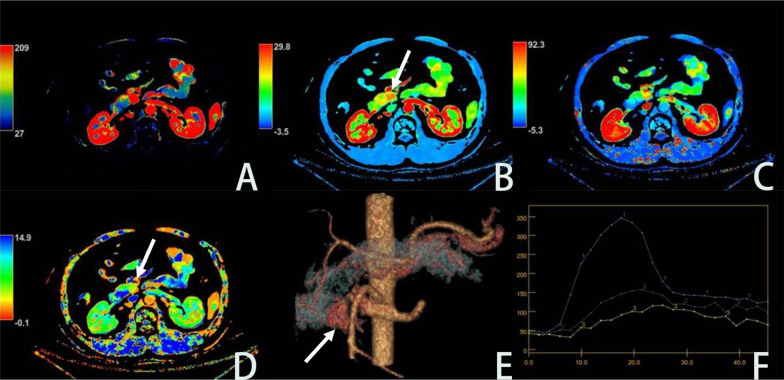
Male, 38 years old, weight 70 kg, the lesion of pNET is located uncinate process of pancreas, **A**–**D** are BF, BV, PS and MTT images, respectively. The BF, BV and PS color scale of the lesion is higher than those of the adjacent normal pancreatic parenchyma, and the MTT color scale is lower than the normal parenchyma. **E** shows the VR image for the lesion. F shows an image of the lesion displaying the perfusion curve, where line 1 is the aorta, line 2 is the lesion and line 3 is the normal parenchymal curve

**Fig. 3:**
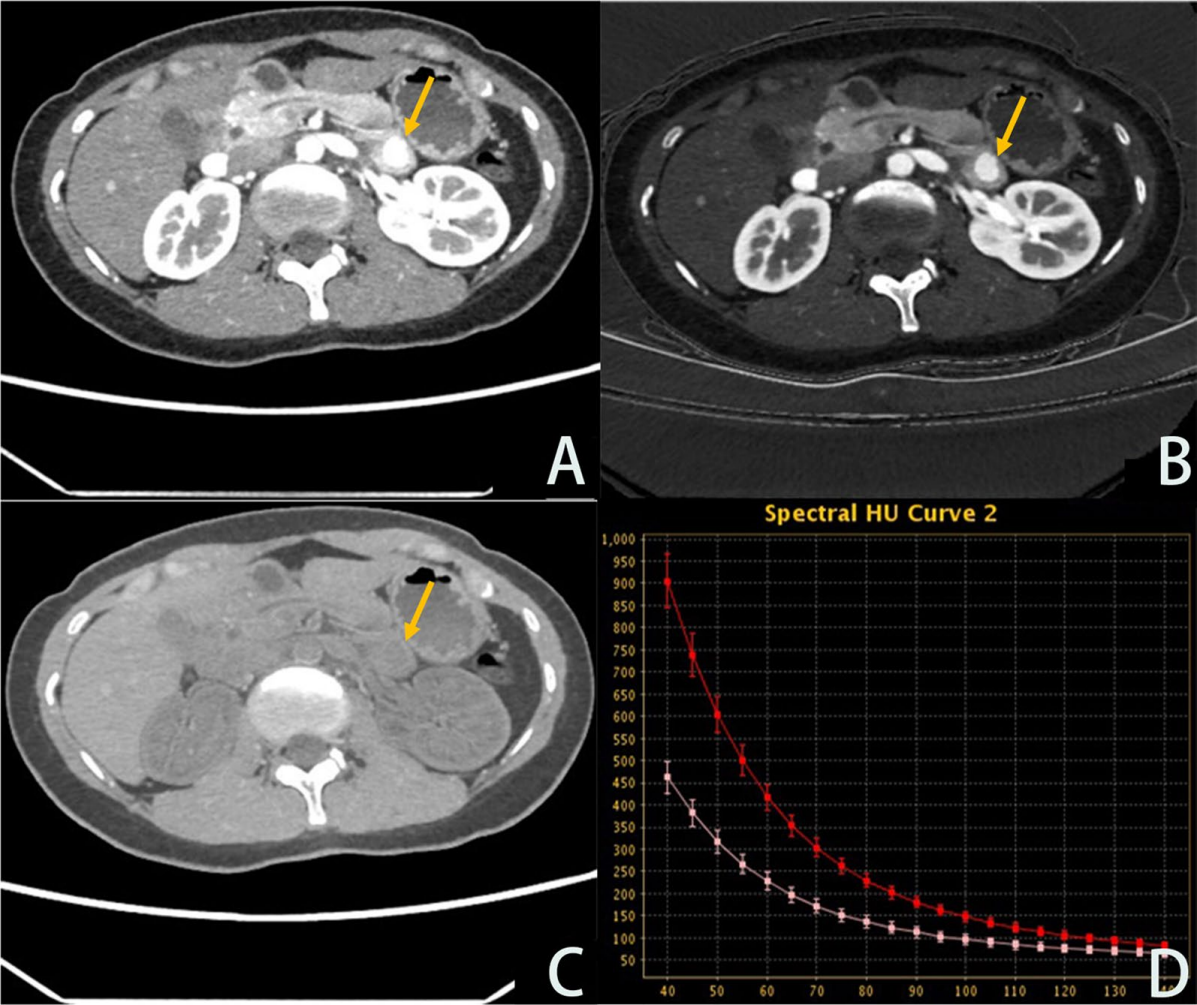
Female, 30 years old, weight 60 kg, the lesion of pNET is located body and tail of pancreatic. **A**–**C** are the arterial phase energy spectrum enhancement images 70 keV images, iodine-based images, and water-based images, respectively, with the lesion enhanced to a higher degree than the adjacent parenchyma; **D** is the arterial phase energy spectrum curve, in which the red line is the lesion curve and the pink line is the normal parenchyma curve

The perfusion parameters BF (ml/min^−1^·100 g^−1^), BV (ml/100 g) and MSI of the pNET normal parenchyma group were lower than those of the lesion group, PS (ml/min^−1^·100 g^−1^), MTT (s), TTP (s), Tmax (s) in the normal parenchyma group were higher in the lesion group, and the difference was statistically significant (both *p* < 0.05), but there was no significant difference in IRF T0 between the two groups (*p* > 0.05) (Table 3, Figs. 2, 3).

**Table 3:** Comparison of perfusion parameters between lesions group and normal parenchyma group

Group/infusion parameters	Normal parenchyma group	Lesions group	*p* value
BF	128.98 ± 69.33	238.24 ± 133.58	0.000
BV	16.99 ± 3.92	22.67 ± 6.54	0.000
PS	7.43 ± 6.89	3.28 ± 1.79	0.000
TTP	19.12 ± 4.42	16.48 ± 2.37	0.000
Tmax	5.43 ± 1.69	4.11 ± 1.78	0.000
MSI	6.39 ± 1.47	10.60 ± 3.37	0.000
MTT	9.56 ± 4.49	6.98 ± 3.52	0.007
IRF T0	1.06 ± 0.09	0.94 ± 0.10	0.860

*t* value, respectively: 3.609, 3.662, − 2.70, − 5.712, − 6.795, 12.874, − 2.695, 0.182

### Comparison of perfusion parameters among different grades of pNETs

Forty-eight lesions of pathologically confirmed pNETs were included in this cross-grade analysis, including 27 cases of grade G1, 17 cases of grade G2 and 4 cases of grade G3. Due to the limited cases and less clinical challenges in diagnosing G3 tumors [8–10], we only compared the 27 G1 and 17 G2 tumors. The median age of patients in group G1 and group G2 was 47.5 years and 51 years, respectively, without any significant difference (*p* > 0.05); the diameter of the lesions were 16.24 ± 4.89 mm and 19.76 ± 4.54 mm, respectively, without any significant difference (*p* > 0.05); perfusion parameters including BV, MTT, PS, TTP, MSI and *T*max, did not show any significant difference (*p* > 0.05), but the BF and IRF T0 values in G2 group were significantly higher than those of G1 group (Table 4).

**Table 4:** Comparison of perfusion parameters between pNETs G1 and G2 groups

Grade/perfusion parameters	G1(27 cases)	G2(17 cases)	*p* value
BV	20.37 ± 1.32	21.04 ± 1.17	0.708
PS	5.24 ± 0.53	5.83 ± 0.74	0.681
MTT	6.41 ± 0.47	6.02 ± 0.78	0.750
TTP	16.48 ± 0.45	18.17 ± 0.52	0.682
Tmax	3.73 ± 0.28	4.51 ± 0.35	0.587
MSI	10.62 ± 0.57	9.04 ± 0.62	0.140
BF	224.72 ± 77.95	287.63 ± 89.07	0.032
IRF T0	0.66 ± 0.10	1.56 ± 0.25	0.046

### Analysis of spectral parameters between pNETs lesions and normal pancreatic parenchyma

CT values of the lesions under each single energy in the arterial phase of pNETs were statistically higher than those of the adjacent normal pancreatic parenchyma (*p* < 0.05); while the CT values in the venous phase were similar to the surrounding normal pancreatic parenchyma (Table 5). The IC (µg/cm^3^) and NIC (µg/cm^3^) of pNETs lesions in the arterial and venous phases were all statistically higher than those of the adjacent normal pancreatic parenchyma, and WC (µg/cm^3^) values of pNETs lesions only in the arterial phase showed a significant increase in comparison with the normal parenchyma (Table 6).

**Table 5:** Comparison of CT values of each single energy keV and IC, WC and NIC values in the arterial phase between normal pancreatic parenchyma group and the lesions group

	Normal parenchyma group	Lesions group	*p* value
60 keV	163.73 ± 60.80	273.11 ± 28.36	0.000
70 keV	125.18 ± 44.17	206.42 ± 20.96	0.000
80 keV	99.64 ± 34.24	167.27 ± 18.07	0.000
90 keV	83.06 ± 27.67	139.50 ± 15.79	0.000
100 keV	71.03 ± 23.35	120.74 ± 16.37	0.000
110 keV	62.56 ± 20.34	106.58 ± 16.28	0.002
120 keV	56.65 ± 18.30	94.35 ± 15.22	0.007
130 keV	51.91 ± 16.95	87.46 ± 15.01	0.026
140 keV	48.32 ± 15.71	80.22 ± 14.29	0.032
IC	37.71 ± 14.68	82.31 ± 33.71	0.000
NIC	0.34 ± 0.17	0.76 ± 0.50	0.000
WC	1031.38 ± 9.76	1070.7 ± 72.32	0.004

**Table 6:** Comparison of CT values of each single energy keV and IC, WC and NIC values in the venous phase between normal pancreatic parenchyma group and the lesions group

	Normal parenchyma group	Lesions group	*p* value
60 keV	114.04 ± 19.81	117.54 ± 9.07	0.896
70 keV	89.73 ± 15.75	95.92 ± 8.71	0.182
80 keV	74.74 ± 13.18	78.80 ± 7.76	0.481
90 keV	63.83 ± 11.74	67.27 ± 6.89	0.461
100 keV	56.15 ± 10.80	58.66 ± 6.12	0.577
110 keV	51.30 ± 9.67	52.75 ± 5.94	0.743
120 keV	47.79 ± 9.07	48.75 ± 5.53	0.967
130 keV	44.70 ± 8.90	45.71 ± 5.17	0.923
140 keV	42.20 ± 9.12	42.99 ± 5.06	0.954
IC	24.13 ± 3.74	37.26 ± 26.28	0.014
NIC	0.62 ± 0.08	0.78 ± 0.20	0.001
WC	1032.57 ± 9.76	1051.91 ± 45.62	0.120

### Radiation dose analysis

The average effective radiation dose received by patients during the whole scanning process was 19.24 ± 3.21 mSv; the effective radiation dose during the perfusion combined energy spectrum enhanced scanning process was 17.51 ± 2.18 mSv, and the radiation dose of the upper abdominal plain scan was 2.15 ± 0.51 mSv.

## Discussion

In this study, we reported clinical evidence of applying energy spectrum combined with perfusion CT scan in imaging the entire pancreatic tissue with a relative low radiation dose in detecting 96.6% pNETs in the 66-patient cohort. CT examination has become a common method for the diagnosis of pNETs, but conventional enhanced CT scan has certain limitations, especially for detecting pNETs with small size or functional pNETs which show atypical enhancement pattern. CT perfusion imaging is a functional imaging in quantifying changes of blood perfusion in local tissue. It is a great technology for the diagnosis of hypervascular pNETs lesions, but limited on small field of examination and high radiation dose. Multi-functional dynamic helical CT can achieve multi-layer dynamic scanning and increase the Z-axis scanning range; however, its time resolution is sacrificed, which significantly reduce the diagnostic accuracy. Clearly, our study demonstrated significant advantages of combining energy spectrum with perfusion CT scan in the application of diagnosing pNETs.

CT energy spectrum imaging uses high and low dual-energy X-rays to perform two rotation scanning imaging and uses the different absorption of X-rays produced by substances to provide more image information than conventional CT [11]. In addition to images of the base substances, images of different single energies (40~140 keV) can also be obtained, which improves image quality; meanwhile, CT energy spectroscopy can perform instantaneous keV switching, which can suppress beam hardening artifacts and reduce radiation dose [12]. Based on these features, multi-energy spectral imaging can improve soft tissue contrast and provide qualitative and quantitative diagnosis of small lesions that are difficult to characterize by conventional CT. In this study, the detection rate of pNETs lesions was 96.6% in the arterial phase, which was much higher than the conventional pancreatic perfusion scanning (82.8%). In addition, the tube voltage was 80 kVp and 140 kVp with instantaneous switch; the tube current was automatically adjusted, and a 40% AsiR-V calculation method was used to reduce the radiation dose to 17.51 ± 2.18 mSv, about 10% lower than the 19.47 ± 4.71 mSv of pancreas perfusion CT in our own study, and lower than the previous report by Zhu et al. on perfusion CT too [9].

Previous studies on diagnostic CT imaging for pNETs were mostly centered on individual examination technique, but this study is innovated by combining spectral CT with perfusion scan, a single scan simultaneously obtaines tumor enhancement characteristics, perfusion parameters and energy spectrum parameters. Most pNETs in our study showed rich blood supply, and the solid part of the tumor was obviously enhanced. In terms of perfusion parameters, pNETs in our cohort showed hyper-perfusive characteristics, that differed from adjacent normal parenchyma. The perfusion parameter BF and BV of pNETs were 238.24 ± 133.58 ml/min^−1^·100 g^−1^ and 22.67 ± 6.54 ml/100 g, remarkably higher than those of adjacent pancreatic parenchyma, and consistent with the results of Choi et al. [13–15], indicating the abnormal hypervascularity of the tumors to sustain their growth. We also identified a lower perfusion parameter MTT in pNETs than that in the adjacent parenchyma, which may be related to the abnormal proliferation of microvascular inside tumors [14]. In addition, the perfusion parameter PS was significantly lower in pNETs than in normal pancreatic tissue which is consistent with the previous report [16]. We postulate that the abnormal proliferation of tumor and interstitial tissue would increase the pressure in extravascular space in compressing blood vessels, which causes the slowdown of contrast agent to pass through the extravascular tissue space. We also noticed the change of the time-density curve of the tumors. The lesions were mostly developed in the arterial phase between 19.4 and 24.1 s, which is delayed in comparison with the conventional arterial enhanced scan time that is earlier than 19.4 s. This result may implicate potential significance for the display of suspected pNETs, but further investigation is needed [17]. The dual-phase energy spectrum enhanced images showed that the CT values of pNETs lesions at different single energies in the arterial phase were significantly higher than those of adjacent normal pancreatic parenchyma, but no difference between the two in the venous phase, indicating that pNETs tumors are rich in blood, and suggesting an advantage of lesion detection in the arterial phase. In addition, the base substance analysis showed that the values of IC, WC and NIC of lesions in arterial and venous phases were statistically higher than those of adjacent normal parenchyma. It was noticeable that the CT energy spectrum curve of the lesion was higher than that of the normal parenchyma. With the increase in single energy keV, the difference between the two was smaller, indicating that single energy images of lower energy range between 60 to 90 keV can be used for better diagnosis of lesions if only dual-phase energy spectrum scanning of the pancreas is performed.

All pNETs are considered to have malignant potential according to the 2017 WHO standard, and are divided into three grades: G1, G2 and G3 based on mitotic cell number and Ki-67 index. Accurate preoperative grading of pNETs is critical for surgery planning, which is the first-line choice for pNETs treatment. In our study between the G1 and G2 groups, perfusion parameter BF and IRF T0 in G2 pNETswere significantly higher indicating higher blood flow, which may be related to the abnormal proliferation of tumor vascular structures. However, due to the small number of samples of different grades of tumors, the energy spectrum parameters were not compared between different grades. In addition, we are aware of several other limitations of our study, (1) This study was a single-center study; (2) Although the data were measured by two radiologists, the Kappa value was not calculated, there might be certain bias or error, thus the results need further verification. However, the final diagnosis was agreed after consultation with a senior radiologist when different opinions occurred. Overall, our study indicates that the spectral CT combined with perfusion scan with GE Revolution CT improves the detection of pNETs according to morphological features, perfusion parameters and energy spectrum characters with a relatively small radiation dose.

## Data Availability

Due to privacy regulations, the clinical data collected in this study are not deposited in a public registry, but the data can be made available via a request to the corresponding author.

## Abbreviations

BF: Blood flow
BV: Blood volume
CT: Computerized tomography
DLP: Dose-length product
ED: Effective dose
IC: Iodine concentration
IRF T0: Idoine remaining function time 0
MSI: Mean slope of increase
MSI: Mean slope of increase
MTT: Mean transit time
NIC: Normalized iodine concentration
pNETs: Pancreatic neuroendocrine tumors
PS: Permeability of surface
Tmax: Transit time to pulse peak
TTP: Time to peak
WC: Water concentration

## References

[1] Jeune F, Taibi A, Gaujoux S (2020). Update on the surgical treatment of pancreatic neuroendocrine tumors. Scand J Surg.

[2] Kulke MH, Ruszniewski P, Van Cutsem E (2019). A randomized, open-label, phase 2 study of everolimus in combination with pasireotide LAR or everolimus alone in advanced, well-differentiated, progressive pancreatic neuroendocrine tumors: COOPERATE-2 trial. Ann Oncol.

[3] Vaghaiwalla T, Keutgen XM (2020). Surgical management of pancreatic neuroendocrine tumors. Surg Oncol Clin N Am.

[4] Wang H, Lin Z, Li G (2020). Validation and modification of staging systems for poorly differentiated pancreatic neuroendocrine carcinoma. BMC Cancer.

[5] Belousova E, Karmazanovsky G, Kriger A (2017). Contrast-enhanced MDCT in patients with pancreatic neuroendocrine tumours: correlation with histological findings and diagnostic performance in differentiation between tumour grades. Clin Radiol.

[6] Salahshour F, Mehrabinejad MM, Zare Dehnavi A (2020). Pancreatic neuroendocrine tumors (pNETs): the predictive value of MDCT characteristics in the differentiation of histopathological grades. Abdom Radiol (NY).

[7] Dromain C, Déandréis D, Scoazec JY (2016). Imaging of neuroendocrine tumors of the pancreas. Diagn Interv Imaging.

[8] Lee DW, Kim MK, Kim HG (2017). Diagnosis of pancreatic neuroendocrine tumors. Clin Endosc.

[9] Zhu L, Xue H, Sun H (2017). Insulinoma detection with MDCT: is there a role for whole-pancreas perfusion?. AJR Am J Roentgenol.

[10] Lv P, Lin XZ, Li J, Chen K (2011). Differentiation of small hepatic hemangioma from small hepatocellular carcinoma: recently introduced spectral CT method. Radiology.

[11] Patel BN, Thomas JV, Lockhart ME, Berland LL, Morgan DE (2013). Single-source dual-energy spectral multidetector CT of pancreatic adenocarcinoma: optimization of energy level viewing significantly increases lesion contrast. Clin Radiol.

[12] Pelgrim GJ, van Hamersvelt RW, Willemink MJ (2017). Accuracy of iodine quantification using dual energy CT in latest generation dual source and dual layer CT. Eur Radiol.

[13] Choi TW, Kim JH, Yu MH, Park SJ, Han JK (2018). Pancreatic neuroendocrine tumor: prediction of the tumor grade using CT findings and computerized texture analysis. Acta Radiol.

[14] D'Onofrio M, Cingarlini S, Ortolani S (2017). Perfusion CT changes in liver metastases from pancreatic neuroendocrine tumors during everolimus treatment. Anticancer Res.

[15] Xue HD, Jin ZY, Liu W, Wang Y, Zhao WM (2006). Perfusion characteristics of normal pancreas and insulinoma on multi-slice spiral CT. Zhongguo Yi Xue Ke Xue Yuan Xue Bao.

[16] Almeida RR, Lo GC, Patino M, Bizzo B, Canellas R, Sahani DV (2018). Advances in pancreatic CT imaging. AJR Am J Roentgenol.

[17] Yao JC, Phan AT, Hess K (2015). Perfusion computed tomography as functional biomarker in randomized run-in study of bevacizumab and everolimus in well-differentiated neuroendocrine tumors. Pancreas.

